# Identifying and mapping individual medicinal plant *Lamiophlomis rotata* at high elevations by using unmanned aerial vehicles and deep learning

**DOI:** 10.1186/s13007-023-01015-z

**Published:** 2023-04-01

**Authors:** Rong Ding, Jiawei Luo, Chenghui Wang, Lianhui Yu, Jiangkai Yang, Meng Wang, Shihong Zhong, Rui Gu

**Affiliations:** 1 School of Pharmacy, Chengdu University of Traditional Chinese Medicine, Chengdu, 611137 China; 2 West China Biomedical Big Data Center, West China Hospital/West China School of Medicine, Sichuan University, Chengdu, 610044 China; 3 Chengdu Pushi Pharmaceutical Technology Co., Ltd, Chengdu, 611100 China; 4 Institute of Geological Survey of Sichuan Provincial, Chengdu, 610081 China; 5 School of Pharmacy, Southwest Minzu University, Chengdu, 610041 China; 6 School of Ethnic Medicine, Chengdu University of Traditional Chinese Medicine, Chengdu, 611137 China

**Keywords:** *Lamiophlomis rotata*, Orthomosaic, Medicinal plant detection, Medicinal plant mapping, Yield prediction, Deep learning

## Abstract

**Background:**

The identification and enumeration of medicinal plants at high elevations is an important part of accurate yield calculations. However, the current assessment of medicinal plant reserves continues to rely on field sampling surveys, which are cumbersome and time-consuming. Recently, unmanned aerial vehicle (UAV) remote sensing and deep learning (DL) have provided ultrahigh-resolution imagery and high-accuracy object recognition techniques, respectively, providing an excellent opportunity to improve the current manual surveying of plants. However, accurate segmentation of individual plants from drone images remains a significant challenge due to the large variation in size, geometry, and distribution of medicinal plants.

**Results:**

In this study, we proposed a new pipeline for wild medicinal plant detection and yield assessment based on UAV and DL that was specifically designed for detecting wild medicinal plants in an orthomosaic. We used a drone to collect panoramic images of *Lamioplomis rotata* Kudo (LR) in high-altitude areas. Then, we annotated and cropped these images into equally sized sub-images and used a DL model Mask R-CNN for object detection and segmentation of LR. Finally, on the basis of the segmentation results, we accurately counted the number and yield of LRs. The results showed that the Mask R-CNN model based on the ResNet-101 backbone network was superior to ResNet-50 in all evaluation indicators. The average identification precision of LR by Mask R-CNN based on the ResNet-101 backbone network was 89.34%, while that of ResNet-50 was 88.32%. The cross-validation results showed that the average accuracy of ResNet-101 was 78.73%, while that of ResNet-50 was 71.25%. According to the orthomosaic, the average number and yield of LR in the two sample sites were 19,376 plants and 57.93 kg and 19,129 plants and 73.5 kg respectively.

**Conclusions:**

The combination of DL and UAV remote sensing reveals significant promise in medicinal plant detection, counting, and yield prediction, which will benefit the monitoring of their populations for conservation assessment and management, among other applications.

**Supplementary Information:**

The online version contains supplementary material available at 10.1186/s13007-023-01015-z.

## Background

Medicinal plants are valuable sources of herbal goods worldwide. More than one-tenth of plant species are utilized in health products and medicines. In particular, the herbal formulas of traditional Chinese medicine are widely used in the treatment of COVID-19 in mainland China [1]. China is one of the most species-diverse countries in the world, with over 10,000 medicinal plant species [2, 3]. The rapid rise of the Chinese medicine market has resulted in medicinal plant resources disappearing at a high speed [4–6], resulting in the rapid expansion of medicinal plant cultivation. However, 70% of commonly used herbal medicines continue to rely on natural resources. Therefore, obtaining information on the status of wild medicinal plant resources, such as plant density, area or several individuals per hectare, is one of the most important yield components in medicinal plants [7, 8]. Deep learning (DL) and unmanned aerial vehicles (UAVs) are technical and methodological advancements that strongly contribute to plant identification shifts. Examples include crop yield prediction [9, 10] and weed mapping [11, 12] in precision agriculture, invasive species identification [13, 14], species detection and vegetation classification [15, 16] in ecological aspects, and area calculation in cultivated herbal medicine [17, 18].

Identifying and counting of medicinal plant individuals is a critical task for yield assessment and resource protection when dealing with wild medicinal plant resources. Usually, wild medicinal plants are diversely and irregularly distributed. Furthermore, medicinal plants are distributed over several hectares, leading to an extremely large obtained image. This situation results in great difficulties in distinguishing wild medicinal plants. High-resolution imagery was captured by utilizing UAVs flying at low altitudes equipped with red–green–blue (RGB) cameras. Plants can be detected in digital images either by manually detection or by using automatic analysis techniques. However, in the calculation of medicinal plant yields and mapping of medicinal plants across the entire distribution region, the target plants must be correctly identified and segmented from the high-resolution orthomosaic generated by UAV photogrammetry. After capturing and correcting images in the presence of camera tilt and relief displacement, the product generated from the study area is called an orthomosaic. This approach is designed to obtain a general image of that study area on a single scale. Therefore, the orthomosaic can be used to visually assess the field, which will provide contextual information about its state and quality [7]. Orthomosaics have been successfully used to map the distribution of herbaceous species, but the ecological background is often too extremely simple or the plants are large and can be quantified using vegetation mapping techniques, and only a few cases of small target plants mapping exist [19, 20]. Until now, few studies have attempted to identify or quantify herbaceous species from UAV imagery [21, 22], particularly medicinal plants. The success of these studies usually depends on the main color difference between the size of target plants and background vegetation.

For the detection of plants in orthomosaics, different model algorithms have been applied in recent studies for counting and mapping invasive species, crops and other species. While numerous studies have shown the ability to detect individual plant species from UAV imagery, most of these plants are shrubs, trees, or tall herbs that are easily identified by the model. For example, James and Bradshaw [16] integrated DL in UAV remote sensing to perform real-time detection of invasive plant shrubs of the Hakea genus, which are 3–5 m tall in the top view of the ecological community and are well suited for real-time detection by UAVs. Zhang et al. [23] identified and mapped frailejones in high-altitude ecosystems by using UAV images, which have plant sizes ranging between 10 and 15 m, are aggregated population species within their distribution area and are also easily identified using UAV. Applications in agriculture are also aimed at the more cultivated neatly patched crop classifications or weeds of larger strains. Therefore, the present UAV recognition of plant objects is primarily for plants of larger size. Traditional machine learning or DL can be used to achieve a high identification effect.

Compared with studies on other plants and wild medicinal plants, detecting medicinal plants requires delineation of individual medicinal plants with multiple leaves. In addition, the yield of medicinal plants in the acquired orthomosaic of drones must also be counted and calculated. In recent years, many studies have shown that mapping and counting other plant species based on different UAV imagery attain moderate results. Examples include potato and lettuce crop counting [24], rice seedling counting [25], sorghum spike detection and counting [26], citrus counting [27]. However, most of these cases are for standardized crops or target fruit trees of larger size, which poses a great challenge for the identification and enumeration of wild medicinal plants, such as complex growth background environment, scattered distribution of target species with different sizes, interference of similar species [28].

The resolution required for individual plant detection and segmentation has stringent requirements for both UAV and DL. Researchers and government managers can monitor resources and predict the yield of medicinal plants by identifying and segmenting each plant. A possible approach is to calculate the yield of the distribution area by calculating the area of the aboveground part of the medicinal plants. Current DL has been able to calculate the aboveground biomass of plants from UAV imagery, which extracts information about targets from an image by separating out the target plants [29]. For example, the development of semantic segmentation models based on convolutional neural networks (CNNs) and the popularization of fully connected networks (FCNs) [30] have considerably contributed to the semantic segmentation of remote sensing images. CNN is designed to analyze spatial pattern analysis. CNNs have been increasingly and widely used to detect plant species in the image processing field with state-of-the-art performance [31–34]. Some of the most popular architectures are the Region CNN (RCNN) [35], Fast RCNN [35, 36], Faster RCNN [37], Mask RCNN (a network that combines Faster RCNN and FCN) [38], and single-shot multibox detector [39]. For example, Nie et al. [40] used an improved Mask R-CNN to detect and segment a ship from remote sensing images. In addition, image segmentation can also be used to accurately measure plant biovolume. Safonova et al. [41] segmented olive tree crowns and shadows to estimate the biovolume of individual trees by Mask R-CNN and UAV images. To monitor the growth status of maize plants, Lu et al. [42] proposed the TasselNet network to achieve robust in-field counting of maize tassels. And, there is also a study the stand counting of maize plants from UAV images [43]. Moreover, image segmentation can be used for crop and weed segmentation [44–46], medical image segmentation [47, 48], and other applications [49, 50]. Thus, the Mask R-CNN model was used more often than other models for similar problems.

In this study, we used Mask R-CNN on ultrahigh resolution UAV-based imagery to properly identify and map individuals of a specific herbaceous species growing close to the ground in natural complex environments. In 2015, Girshick [36] proposed a fixed-size pooling layer for regions of interest that can improve the speed of R-CNN, that is, Fast R-CNN. In 2017, He et al. [38] introduced Mask R-CNN, which performs excellently in image classification, semantic segmentation and instance segmentation. Mask R-CNN not only accurately detects the target class and location information in the remote sensing images but also obtains the binary mask for each class instance. The proposed method counts and predicts the yield of medicinal plants on UAV remote sensing and maps the distribution of the species. This study aims to demonstrate the usefulness of the method in the detection of small target individual medicinal plants, solve the ecological survey problems, develop an efficient and fast survey methodology, and create a new technical method that makes it possible to utilize Chinese medicine resources effectively.

Our case study takes as an example the Tibetan medicine *Lamiophlomis rotata* (Benth. ex Hook. f.) Kudo (LR), a perennial medicinal herb endemic to the Qinghai-Tibet Plateau, has been one of the traditional medicines of the Tibetan, Mongolian and Na Xi peoples for thousands of years and is locally known as “Daba” or “Daerba” [51]. LR has the efficacy of hemostasis and alleviating pain and is widely used in the treatment of postsurgical incision pain, bleeding, rheumatism arthralgia, etc. [52, 53]. LR grows in alpine meadows, gravel beaches or riverbanks at altitudes ranging between 2700 and 4500 m above mean sea level. In recent years, owing to their strong demand in the market, LR herbs have encountered severe overexploitation, as medicinal herbs all rely on wild resources. In fact, LR was listed as a first-level endangered Tibetan medicine in 2000. Moreover, LR is an indicator plant for degraded ecosystems of high-altitude grasslands, scrub grasslands and wetlands. Furthermore, LR is under pressure from ecological conservation and degradation of wild resource populations. Population monitoring of LR wild resources is crucial for artificial cultivation, but it is rarely implemented due to the lack of basic research on related field resources [54]. Consequently, yield prediction and species distribution mapping of LR should be urgently performed to further guide germplasm resource utilization, conservation, and breeding strategy development.

## Results

### Classification results

**Fig. 1:**
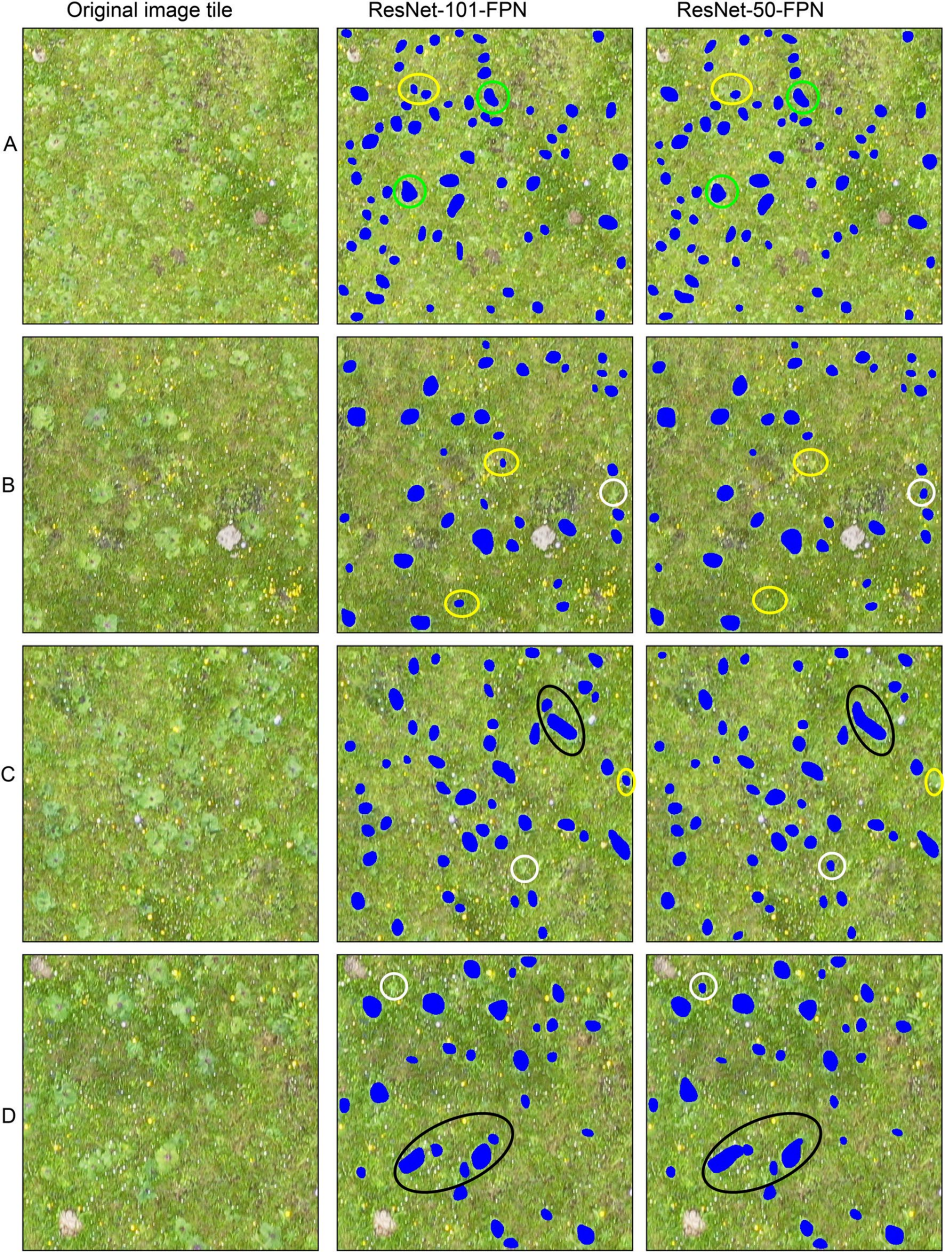
Four subsets (**a**–**d**) of LR classification in S1 by using ResNset-50-FPN and ResNet-101-FPN

**Fig. 2:**
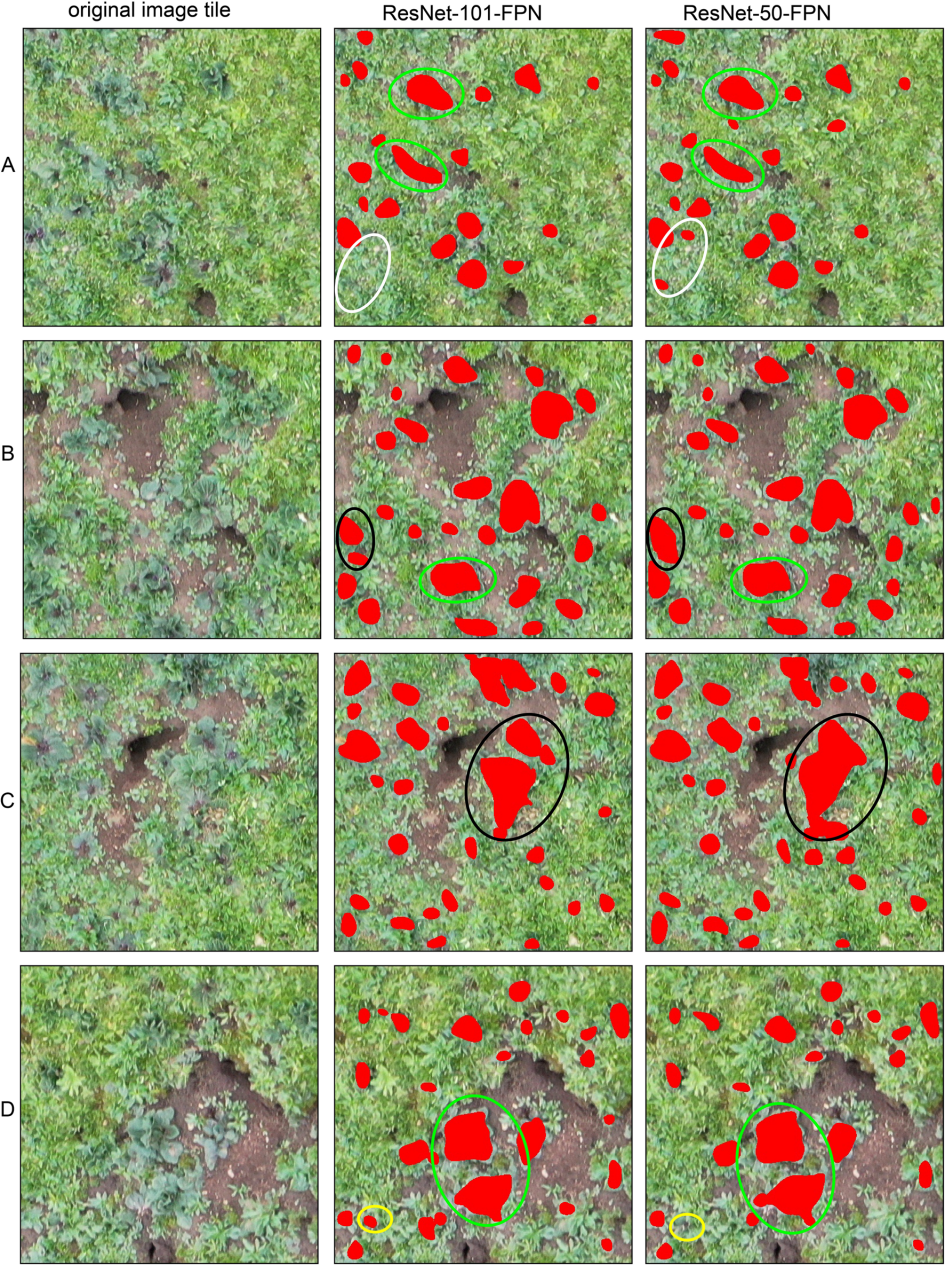
Four subsets (**a**–**d**) of LR classification in S2 by using ResNset-50-FPN and ResNet-101-FPN

Details of the training of the Mask R-CNN network are shown in Additional file 1: Figure S1. The classification results of ResNet-50 and ResNet-101 are shown for 4 image subsets of S1 and S2 (Figs. 1 and 2). Mask R-CNN differentiated each individual LR successfully with accurate boundaries between plants. Obviously, both ResNet-50 and ResNet-101 merged individual LR plants into some continuously distributed patches, such as the green circles of Fig. 1a. ResNet-101 achieved a higher accuracy through the edges of LR (Fig. 1c and d), black circles). Moreover, ResNet-101 can identify smaller LR plants than ResNet-50 cannot (yellow circles). Sometimes ResNet-50 will identify weeds that are similar in color and morphology to the LR, but ResNet-101 will not (white circles). Both ResNet-50 and ResNet-101 delivered a similar and better classification result, while still having difficulty separating neighboring LR plants (Fig. 1, green circles and black circles). By contrast, ResNet-101 can distinguish neighboring LR plants more accurately and with fewer misidentifications, with individual plants differentiated clearly.

Figure 2 shows the classification results for four subsets in S2. Figure 2 shows an area containing LR species within a matrix of bare land, rat holes and grass. As illustrated by Fig. 2a–d, ResNet-101 is also superior to ResNet-50 in identifying both smaller and neighboring LR plants and separating individual plants.

The performance of the two methods differs slightly depending on the morphological appearance of LR. In S2, individual LR plants were often merged into continuous patches by the Mask R-CNN, and the geometric shapes of the classified plants were distorted, such as the green circles and black circles in Fig. 2. However, ResNet-101 still outperformed ResNet-50 in segmenting LR (Fig. 2b and c, black circles). The white circles in Fig. 2a and the yellow circles in Fig. 2d are ignored by ResNet-50 and ResNet-101, respectively.

### Accuracy comparison of instance segmentation

A quantitative assessment of classification accuracy provides further evidence of which method is more effective to use. We conducted 10 experiments with different numbers of randomly selected images from the dataset, as shown in Table 1. For both study sites, ResNet-101 achieved the largest precision for the classification of individual LR in S1 (i.e., 93.46%) and the classification of individual LR in S2 (i.e., 85.21%). ResNet-50, however, achieved the highest recall, F1 and mAP for the validation datasets, overall 1% higher than ResNet-101. Among them, the overall accuracy, detection rate and geometric accuracy results of S1 are higher than those of S2, which may be due to the more complex background and morphology of LR in S2. Among the benchmarks, the best performing approach was ResNet-101, followed in sequence by ResNet-50.

**Table 1:** Accuracy assessment for two methods using precision, recall, F1-score and mAP (IOU = 50)

Study sites	Method	Precision	Recall	F1	mAP
S1	ResNet-50-FPN	92.75 $\pm$ 0.61	98.56 $\pm$ 1.04	97.99 $\pm$ 1.27	97.59 $\pm$ 1.65
	ResNet-101-FPN	**93.46** $\pm$ **0.71**	**98.90** $\pm$ **1.21**	**98.61** $\pm$ **1.40**	**98.32** $\pm$ **1.60**
S2	ResNet-50-FPN	83.89 $\pm$ 0.95	90.43 $\pm$ 1.36	87.61 $\pm$ 1.42	84.98 $\pm$ 1.57
	ResNet-101-FPN	**85.21** $\pm$ **0.74**	**91.38** $\pm$ **1.86**	**88.51** $\pm$ **2.05**	**85.81** $\pm$ **2.26**

The value is the mean $\pm$ standard deviation of 10 experiments

### Evaluating the accuracy of the counting

We first evaluate the counting capability of the Mask R-CNN method. Tables 2 and 3 show the Acc and MAE obtained from each for the S1 and S2 datasets. The results shown do not include any postprocessing methods. According to the results, we observed that the average counting accuracy of ResNet-101 was approximately 7% higher than that of ResNet-50. Therefore, in the case of ResNet-101, the average counting accuracy of S1 was approximately 90%, and the average MAE of the counting was approximately 1.2. However, compared with the findings for S1, the average counting accuracy of S2 was approximately 64%, and the average MAE was approximately 3.7. The reason for the low counting accuracy in S2 was the incorrect identification of weeds as LR plants. Therefore, the complexity of the background environment and the size of the dataset affect the accuracy of the LR identification counts.

**Table 2:** Performance of the Mask R-CNN in S1

	ResNet-101-FPN		ResNet-50-FPN	
	ACC (%)	MAE	ACC (%)	MAE
Fold-1	93.42	1.261	87.18	2.418
Fold-2	93.49	1.219	87.08	2.398
Fold-3	90.82	1.226	83.62	2.395
Fold-4	92.70	1.252	86.21	2.438
Average	92.61	1.240	85.93	2.412

**Table 3:** Performance of the Mask R-CNN in S2

	ResNet-101-FPN		ResNet-50-FPN	
	ACC (%)	MAE	ACC (%)	MAE
Fold-1	65.71	3.719	56.91	4.532
Fold-2	60.41	3.823	51.18	4.453
Fold-3	64.32	3.672	56.31	4.312
Fold-4	68.90	3.579	61.88	4.304
Average	64.84	3.698	56.57	4.400

**Fig. 3:**
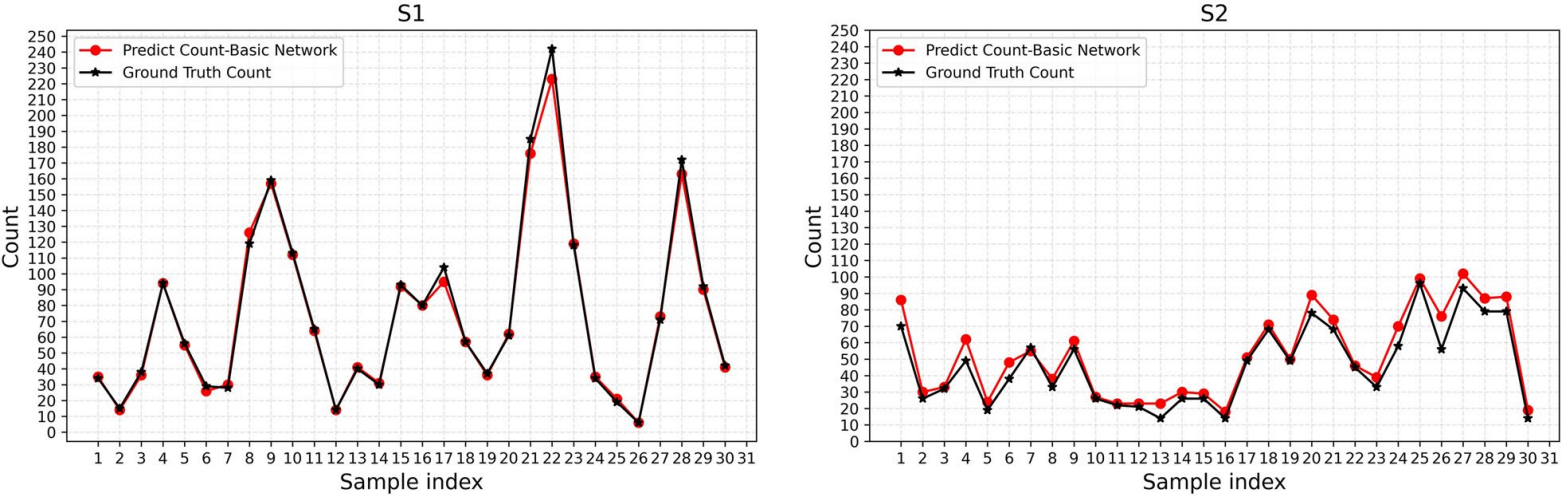
Predicted counted obtained from the Mask R-CNN (ResNet-101) versus ground truth count for each of the 30 plots (2048 × 2048) in the orthomosaic of **S1** and **S2**

Figure 3 shows the predicted counts (using ResNet-101) for each sample and their true counts (using only ResNet-101). The image sources are 30 samples sites of size 2048 × 2048 selected from the orthomosaics of the LR distribution area. In S1, for most images, Mask R-CNN can accurately calculate the number of LR plants, and a few are less than the exact count. In S2, for almost all images, the Mask R-CNN tended to overestimate counts. This overestimation can be regarded as a function of the presence of other “objects,” such as weeds, which are incorrectly assigned a small number of density values by the Mask R-CNN.

**Fig. 4:**
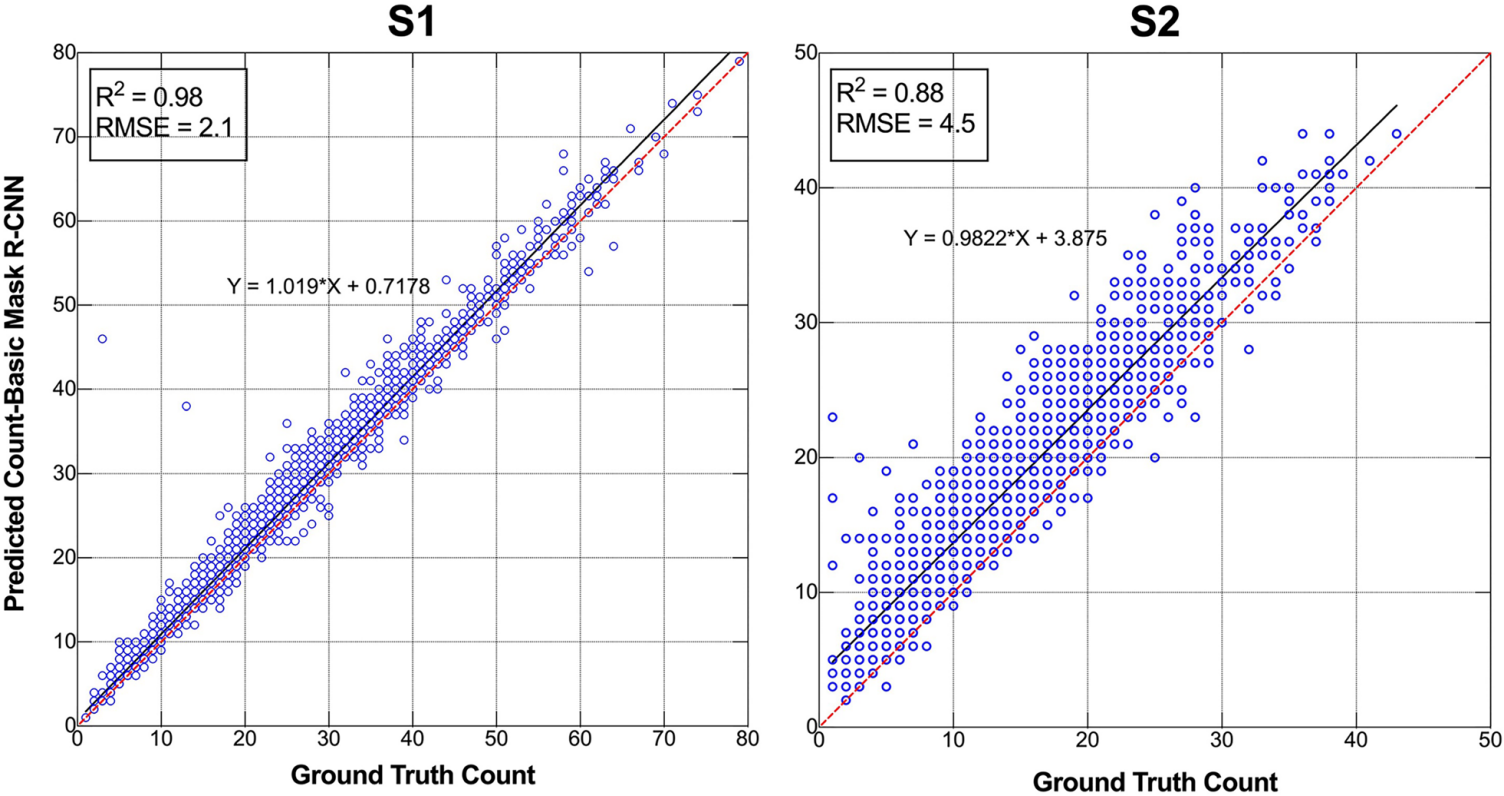
Manual counting versus automatic counting by using the Mask R-CNN (ResNet-101) model at study sites **S1** and **S2**. The red line of 1:1 is the equation of y = x. R^2^ and RMSE represent the coefficient of determination and the root mean square error, respectively

Linear regression analysis was performed on the number of LR plants detected based on manual counts (ground truth) and automatic counts derived from the Mask R-CNN for all images in datasets S1 and S2. As shown in Fig. 4, the Mask R-CNN model performed well in terms of estimating the number of LR plants. In S1, the relationship between manual and automatic LR counts was positive and strong for all images, with R^2^ and RMSE values of 0.98 and 2.1, respectively. In S2, the R^2^ and RMSE were 0.88 and 4.5, respectively. In contrast, the points in S1 are mostly concentrated around y = x, while the points in S2 are mostly located above y = x, indicating more false identification in S2 and better identification in S1. This finding indicates that the Mask R-CNN model can reasonably estimate the number of in-field LR plants.

### Orthomosaic identification and yield calculation results

#### Orthomosaic identification

**Fig. 5:**
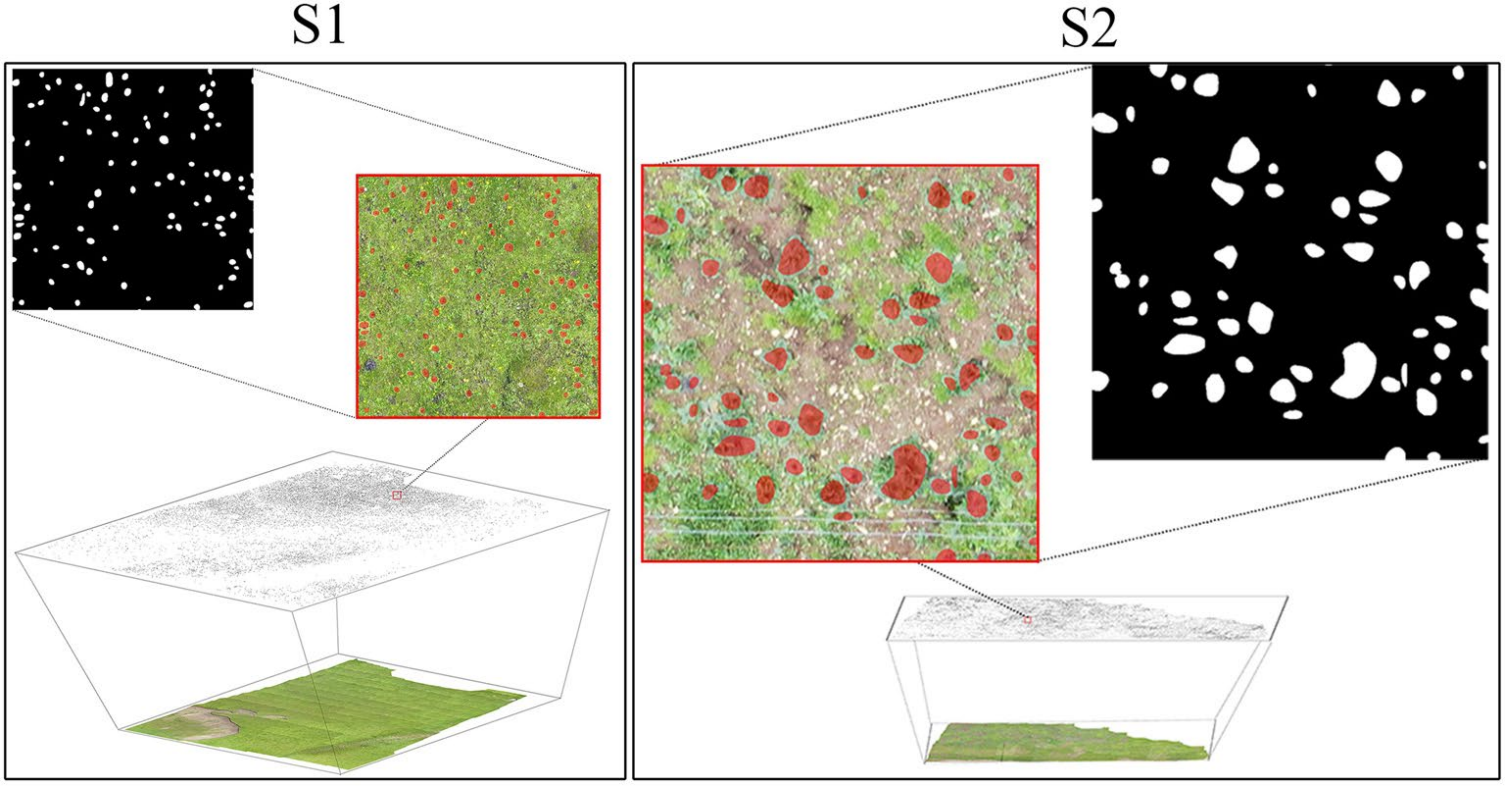
LR identification results in orthomosaic **S1** and **S2**. The black marked points are the locations of the identified LR plants, and the red boxes are enlarged to show the detailed view of the identified image and mask

By identifying LR plants in orthomosaics and creating distribution maps from them, the leaf area and yield of the distribution area were calculated, and the errors of the two methods of calculating yield were compared. Therefore, we used the Mask R-CNN model to perform recognition statistics for LR plants in orthomosaics, and the results are shown in Fig. 5. The statistics are shown in Table 4.

**Table 4:** LR data statistics in orthomosaic

Study sites	Study area (m^2^)	Orthomosaic resolution (m/pixel)	Pixel value (pixel)	Number	Leaf area (m^2^)
S1	9186	0.005	5,323,734	19,376	133.09
S2	2971	0.003	11,453,007	19,129	103.08

### Yield calculation

**Fig. 6:**
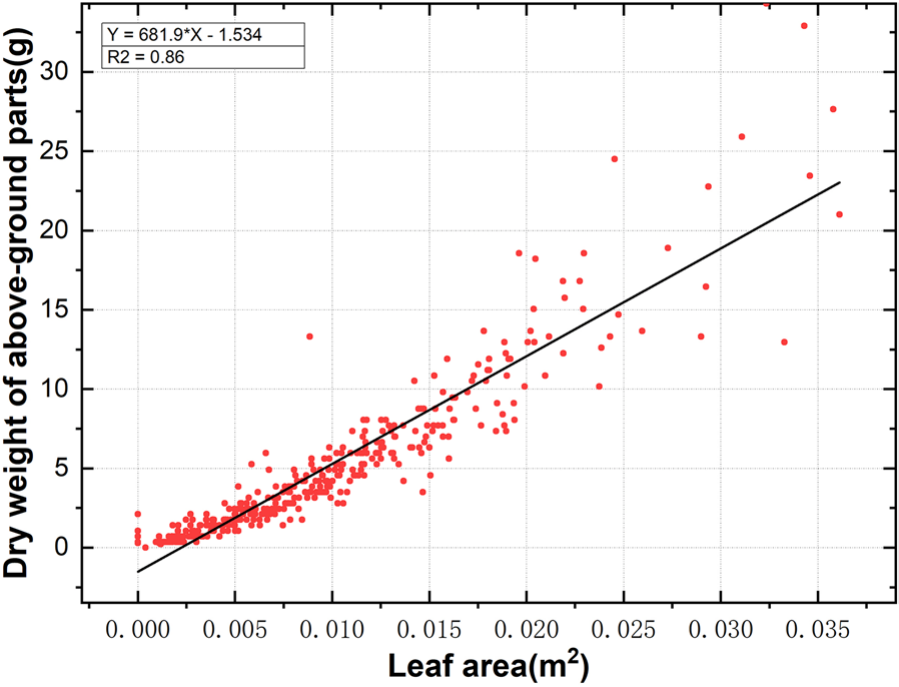
Relationship between LR's dry weight of above-ground parts and leaf area in S1 and S2

Figure 6 shows a significant regression relationship between the dry weight of aboveground parts and leaf area, and the R^2^ of the regression equation was 0.86. The accuracy of this linear model was good, indicating that the yield can be predicted by the leaf area index. The average weight of the LR of S1 was 2.99 g, which is lighter than the average weight of the LR of S2, which was 4.01 g. This result was consistent with the field survey results.

As shown in Table 5, the difference between the yield calculation results of S1 was 32.82 kg, while the yield calculation results of S2 only differ by 6.42 kg. The reason for this may be that the location of S1 is in a hillside location, the relative flight height of the UAV is inconsistent, and the resolution in the orthomosaic is the average resolution, which has a large error with the leaf area calculated by the mask. Therefore, when the land is not flat, the yield calculation error is larger.

**Table 5:** Yield prediction results for LR in S1 and S2

Study sites	Average dry weight (plant/g)	Number	Leaf area (m^2^)	Model-identified leaf area calculation yield (kg)	Model-identified quantity calculation yield (kg)
S1	2.99	19,376	133.09	90.75	57.93
S2	4.01	19,129	103.08	70.29	76.71

## Discussion

In this study, we demonstrate a method for accurately predicting the yield of wild medicinal plants and the number of plants in their distribution area. With image analysis approaches based on DL, an opportunity was provided to identify, map, count, predict yield and monitor endangered medicinal plant individuals in complex and diverse highland ecosystems. The average plant diameter in our study was between 2 and 35 cm. Whenever the target object is extremely small, instance segmentation models face a huge challenge [55, 56]. Using the Mask R-CNN architecture, we propose a model that can output the location and number of all LR plants in an orthomosaic and can perform the instance segmentation task more efficiently, essentially achieving exceptional plant detection and counting. On this basis, yield prediction is also performed. In this task, the goal is to accurately detect plants, estimate the number of plants in each image and show the boundaries of each plant at the pixel level. This finding is contrary to previous related studies, which relied on regression-based models to predict counts in a given area, with mostly regular cultivated plants and without any plant location or plant segmentation [57–59]. Therefore, we have demonstrated its potential in assessing wild medicinal plant reserves by using UAV photographic imagery instead of in-field imagery taken from the ground.

Previous studies have developed algorithms aimed at individual plant segmentation, such as cotton [57] and sorghum heads [60]. However, the targeted plant types and the complexity of the context are not comparable with our work. In terms of the study target, the detection targets were neatly cultivated crop seedlings that were not grown together and could not be evaluated due to a lack of accurately digitized ground-truth masks. Zhang et al. [23] proposed the SS Res U-Net model for semantic segmentation and classification of frailejones by using UAV remote sensing. This work is extremely different from ours, as our goal is not only to generate accurate masks, but also to count and calculate the yield of all target plants under a large-scale area of UAV remote sensing. As a result, our task is much more complex and comprehensive.

Traditional methods of Chinese medicine resource surveys rely on sample surveys and field surveys. Then, the location, area, slope direction, slope, elevation, vegetation, and names and numbers of plants in the sample sites were manually recorded [61]. This method is time-consuming, labor-intensive, and financially demanding, such as the census of Chinese herbal resources in China [62]. To the best of our knowledge, UAV remote sensing has not yet been applied to medicinal plant resource surveys, especially for small target medicinal plants at high altitudes. A direct automated scientific approach to plant instance segmentation is illustrated in this study by combining UAV remote sensing and Mask R-CNN. Infield detection of invasive plant DL has been investigated [16]. Alien vegetation detection in orthomosaic has also been conducted in the field of remote sensing [63] and in real-time applications [64]. In contrast to the approach of previous studies, the features used in this study were learned rather than created. By using DL algorithm, the final quantity and yield predictions were also produced within the orthomosaic. This study provides new techniques and methods for assessing the wild medicinal plant resources on the plateau and improves the accuracy of yield prediction. This method breaks the traditional method of Chinese medicine resource surveys, and the use of UAV remote sensing is also an inevitable trend for future development.

For the DL model presented in this study to be applied in the real world, additional refinement is required. First, terrain-imitation flight may be conducted to avoid large errors in yield prediction results at different flight altitudes. Second, the growing stages, different weather conditions, and times needed to be varied in the datasets to improve the robustness of the model. Third, the model and the UAV are integrated to perform real-time detection in the field. Finally, the predicted results of yield and plant population were not verified by a substantial sample survey.

## Conclusion

In this study, UAV remote sensing and DL are used to segment, count, and predict the yield of medicinal plants on the plateau, combining a straightforward and automated cutting-edge approach. The proposed method is a viable alternative to sample surveys and helps quantify the role of medicinal plants in the ecosystem. We developed an LR dataset from UAV images in this study, and after training with the Mask R-CNN model, the results indicated that the ResNet-101 architecture had higher accuracy metrics than ResNet-50, with a maximum accuracy of 98.9% for S1. According to cross-validation, the average ACC of S1 was 92.61% and MAE was 1.240, and the average ACC of S2 was 64.84% and MAE was 3.698. Furthermore, we used the model to identify LR in large-scale orthomosaics, map the distribution of LR plants, count the number of LR plants in the distribution, and predict the yield by using different calculation methods.

Future research can explore the following directions: First, we will deploy our method to an embedded system on a UAV for online yield estimation of medicinal plant yields, and second, we will continue to enrich the LR dataset, as training data are always key to obtaining good performance, especially with the diversity of such data. Third, the precise localization of wild plants enables us to quantify the role of plant species in the ecosystem; for example, the distribution of LR was analyzed in terms of density, clustering and dispersal, and the information was translated into location, density and hotspot maps to provide advanced visualization tools. Finally, our method can be applied to the identification of other similar medicinal plants, especially in areas difficult for humans to reach at high altitudes.

## Methods

### Study area and target plant

**Fig. 7:**
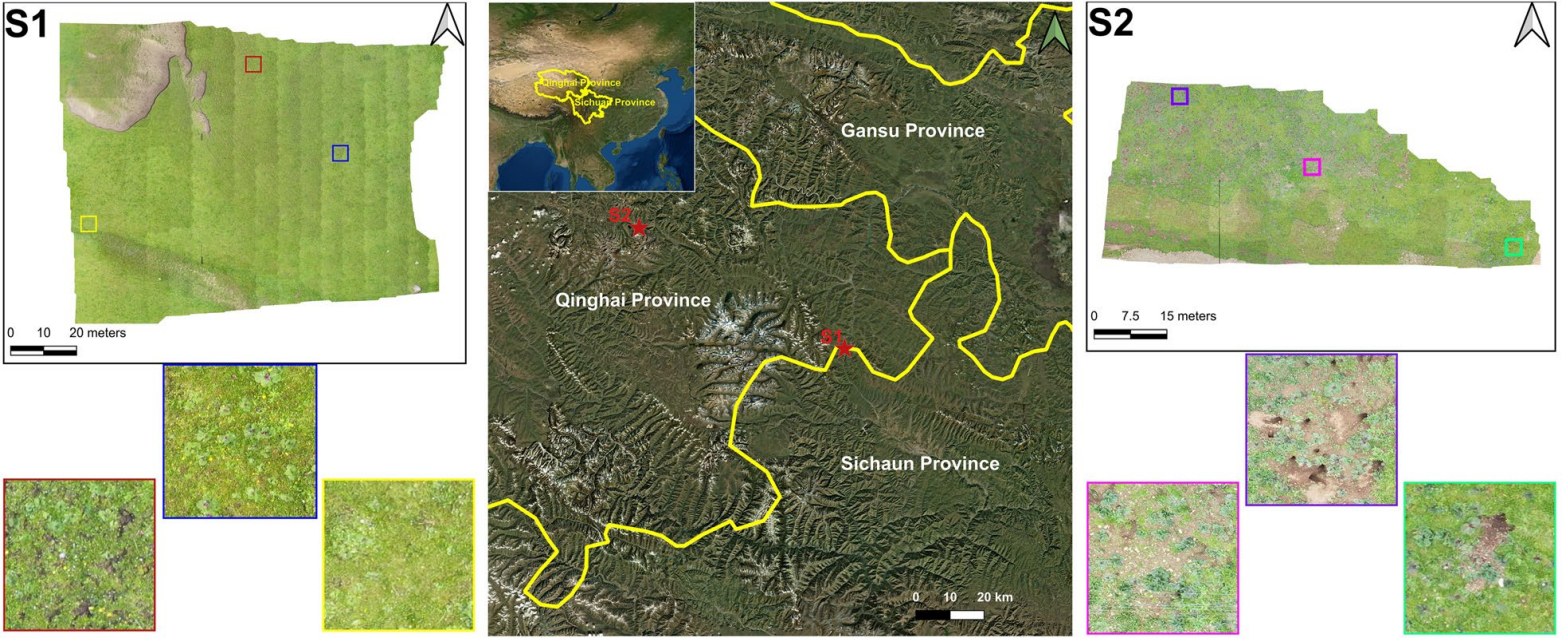
Two study areas in China: Aba County, Sichuan Province (**S1**) and Jiuzhi County, Qinghai Province (**S2**) with typical *Lamiophlomis* species highlighted

The imagery for our case study was collected from two ecosystems within the Ruoke River Ranch, Aba County, Sichuan Province (S1: lat. − 33.21800; long. − 101.46722; elev. − 3774 to 3794 masl), and Jiuzhi County, Qinghai Province (S2: lat. − 33.59244; long. − 100.82794; elev. − 3848 masl) (Fig. 7). LR was located in the top ecological area in the field of view, its size ranged between 2 and 40 cm, it grew close to the ground, and its morphological appearance was different, hindering automatic detection by drone images. The two sample sites were selected to represent most of the ecological environments in which LR grows. Therefore, we focused on mapping the distribution of LR species in the two localities, counting the number of plants, and calculating the yield of the distribution area. Drone images from two sites registered different growth periods and different morphological appearances, including adult plants and young, flowering, unflowered, and clumped plants (Table 6).

**Table 6:** The *Lamiophlomis rotata* species found in the study sites with their descriptions and ground photos of representative plants

Type	Study sites	Descriptions	Plant representative photos
Mature flower	S1	Light green, plant length is approximately 7–35 cm, leaf blade cespitose at the base, mostly 4 leaves, rotate two opposite each other, verticillasters densely arranged in capitula or spikes	
Seedling	S1, S2	Yellowish green—light green, small plant form, not flowering, plant size 2–7 cm	
Mature plant	S2	Dark green, large leaves, often broken and incomplete, plant size 8–30 cm	
Clustered plant	S2	Dark green, growing after the first year of harvest, two to three plants growing together, leaves curled and often squeezed together, plant size 5–30 cm	

LR grows on slopes and hilltops. The type of grassland degradation is moderately degraded grassland, with 80% to 95% Graminaceae, a few Gentianaceae Asteraceae, and other forbs. LR had a high number of seedlings, mature plants and mature flowers, which also included no flowered medium-size plants. The S2 site have many bare ground and rat holes. The type of grassland degradation is heavily degraded grassland with toxic weeds accounting for 60% to 80% and few Graminaceae.

### Dataset collection and annotation

**Fig. 8:**
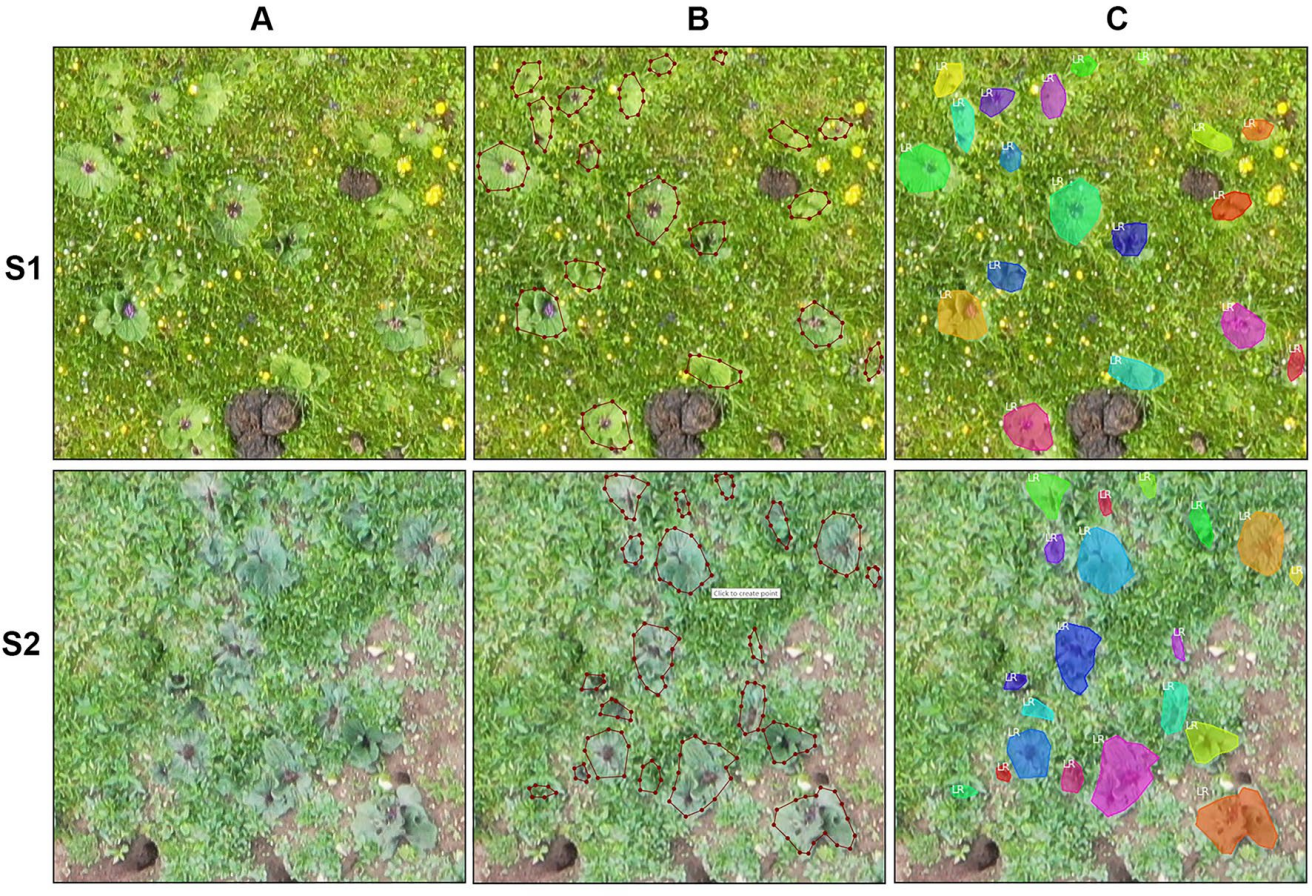
Image annotation by Labelme software. **A** Image tile. **B** Manual labeling. **C** Display after labeling

Aerial photography was obtained by a DJI MAVIC 2 pro drone (DJI Company, Guangdong, China) equipped with a built-in RGB camera with a resolution of 5472 × 3648. Image acquisition was conducted under clear and calm weather conditions in July 2020 because the seasonal LR leaves are dark green, which makes them easily distinguishable from background grass. The flight path was created by the application Pix4D Capture. The flight height was 10 m, and front and side overlaps of 60% were chosen. For the S1 and S2 sites, 353 and 154 images were collected, respectively. The images were aligned using DJI Terra software to produce an orthomosaic and a digital elevation model for each site. For S1 and S2, the stitched images were 22,229 × 20,600 with 0.3 cm average spatial resolution and 30,140 × 15,531 with 0.5 cm average spatial resolution, respectively. We segmented the panorama into several nonoverlapping 512 × 512 images, which ensured that the yield evaluation did not lead to the double counting of yields. Then, each tile was semantically annotated as foreground (target plants) and background (all other land covers), as shown in Fig. 8.

The datasets used are composed of 5700 RGB image tiles, and they contain two types of images: moderately degraded grassland (S1, 3410 images) and severely degraded grassland (S2, 2290 images), with each image being 512 × 512 pixels. The dataset is divided into two subsets with a ratio of 6:4, that is, 3420 pictures as the training set and 2280 pictures as the testing set. Additionally, to enhance the adaptability of our algorithm to the natural environment, the dataset is flipped and enhanced to cover the complex natural environment.

### Mask R-CNN

**Fig. 9:**
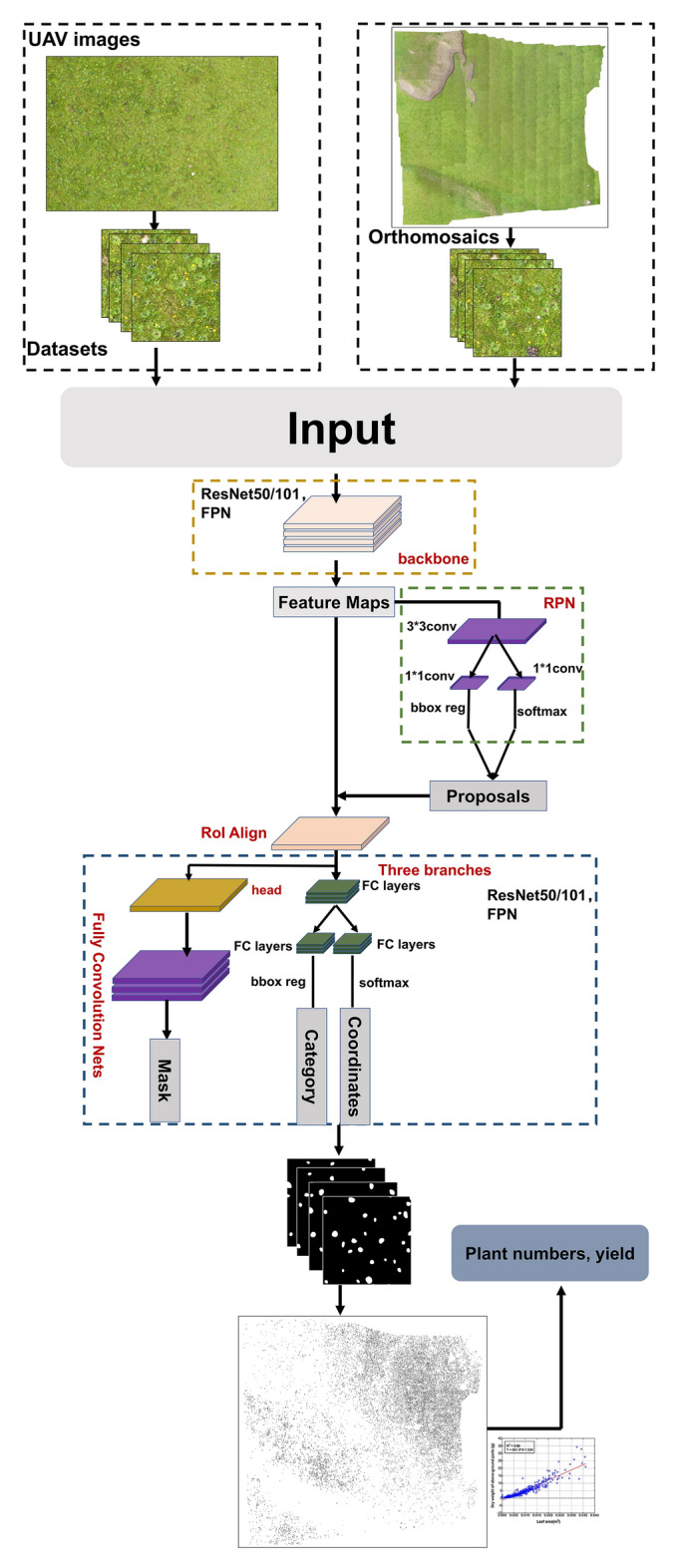
Mask R-CNN network structure and workflow

We identified LR plants from the orthomosaics by using R-CNN masks and subsequently counted and predicted their areas (Fig. 9). Moreover, we also used YOLOACT++ and SOLOv2 networks, but the results were not as good as Mask R-CNN, and the results are shown in Additional file 1: Table S1. Mask R-CNN consists of a region proposal network (RPN), a region-based classification subnetwork, and a semantic segmentation subnetwork. On the basis of the input images, the backbone network creates feature maps; the RPN generates a category-agnostic region of interest (RoI) from the extracted feature maps. Using ROI alignment, we can extract the region feature map by extracting the features that correspond to the ROI. In the detection branch, these region features are used for object classification and bounding box registration. For pixel-level segmentation, the region features from the detection branch of the prediction phase will be updated to the mask branch based on the prediction regions.

In our study, we first extracted the feature map of LR from the cropped image tiles, then the orthomosaic was cropped into image blocks and finally stitched after recognition to obtain the distribution map of LR. The yield of the distribution area was calculated based on the average weight of each plant.

In this work, the ResNet-50 and ResNet-101 are used. This work also compares Mask R-CNN with different backbone networks, and the results are shown in Additional file 1: Table S1. Taking the ResNet-50 as an example, first, the image is cropped to a size of 512 × 512 by using bilinear interpolation and then input to the ResNet network to construct some candidate RoI regions for each element point of each layer of the feature map in the image pyramid, with the long and short dimensions of the RoI regions consisting of two-by-two combinations of (0.5, 1, 2) scales and (8, 16, 32, 64, 128) lengths. The RoI regions in total 15 for a single element point. Coordinate regression and foreground and background classification are performed for each RoI region separately by using the RPN network. For each real region, matching RoI regions are selected, and the regions judged as foreground are ranked according to the intersection of union (IoU) and the output score of the RPN network. The RoI regions with the highest IoU and the highest score are selected as the matching regions for the real regions and classification networks for training.

### Evaluation metrics

#### Model evaluation metrics

As the format of the COCO dataset [65] was adopted in our dataset, four evaluation metrics, mean average precision (mAP50), mean average recall (mAR) and F1-score (balanced score), are used to verify the effectiveness of ResNet-50 and ResNet-101. AP is the average of all the precisions of the IoU threshold. AR represents the average of all recalls with an IoU threshold being in the range of 0.5 to 0.95. The precision, recall and F1-score are calculated as follows:

$$precision= \frac{TP}{TP+FP}$$

$$recall= \frac{TP}{TP+FN}$$

$$F1= 2\times \frac{precision+recall}{precision+recall}$$

where TP represents positive samples correctly identified as positive, FP represents negative samples incorrectly identified as positive, and FN represents positive samples incorrectly identified as negative. Precision reflects the proportion of positive samples predicted to be positive by ResNet, and recall is used to assess how many positive samples were correctly predicted out of the total positive samples.

### Automatic evaluation metrics

The multifold cross validation step was performed to explore the efficacy of the LR automatic counting after determining the final FPN [66, 67]. UAV images of S1 and S2 were randomly assigned to one of four splits. In each cross-validation, we repeated the experiments five times, and the average mean absolute error, accuracy, R^2^, and root mean squared error were used as evaluation metrics:

$$MAE= \frac{1}{n}\sum_{1}^{n}|{t}_{i}-{p}_{i}|$$

$$Acc=\left(1-\frac{1}{n}\sum_{1}^{n}\frac{\left|{t}_{i}-{p}_{i}\right|}{{t}_{i}}\right) \times 100\%$$

$${R}^{2}=1-\frac{\sum_{1}^{n}{({t}_{i}-{p}_{i})}^{2}}{\sum_{1}^{n}{({t}_{i}-\overline{{t }_{i}})}^{2}}$$

$$RMSE= \sqrt{\frac{\sum_{1}^{n}{({t}_{i}-{p}_{i})}^{2}}{n}}$$

where ${t}_{i}$, $\overline{{t }_{i}},$ and ${p}_{i}$ represent the ground truth count for the $i$-th image, the average ground truth count, and the predicted count for the $i$th image, respectively; n represents the number of UAV images in the test set; MAE and Acc quantify the prediction accuracy; and ${R}^{2}$ and RMSE assesses the model performance. The lower the values of MAE and RMSE are, the better the counting performance, while the higher the values of Acc and ${R}^{2}$ are, the better the counting performance.

### Yield calculation

We collected the aboveground part of LR in the field, measured its length and width, brought it back to the laboratory for drying and then weighed it. The dry weight of aboveground parts was predicted by a linear regression model, and the measured length × width was the independent variable. Moreover, we collected 56 and 86 LR plants from the S1 and S2 research areas, respectively, brought them back to the laboratory to dry and weighed them, and calculated the average weight of the LR in S1 and S2. Finally, we used the Mask R-CNN model to predict the LR leaf area and number for S1 and S2 to calculate the LR yield.

## Supplementary Information

**Additional file 1: Figure S1.** Details of the training of the Mask R-CNN network. **Table S1.** Comparison of mA (IOU=50) for Mask R-CNN of different backbone, YOLOACT++ and SOLOv2 models.

## Data Availability

Not applicable.
