# Supplementary material for: Identifying and mapping individual medicinal plant Lamiophlomis rotata at high elevations by using unmanned aerial vehicles and deep learning
Source: Plant Methods. 2023 Apr 1;19:38. doi: 10.1186/s13007-023-01015-z (PMC10066955; doi:10.1186/s13007-023-01015-z)
Supplement: Supplementary file 1 — Additional file 1: Figure S1. Details of the training of the Mask R-CNN network. Table S1. Comparison of mA (IOU=50) for Mask R-CNN of different backbone, YOLOACT++ and SOLOv2 models. [file 13007_2023_1015_MOESM1_ESM.docx]

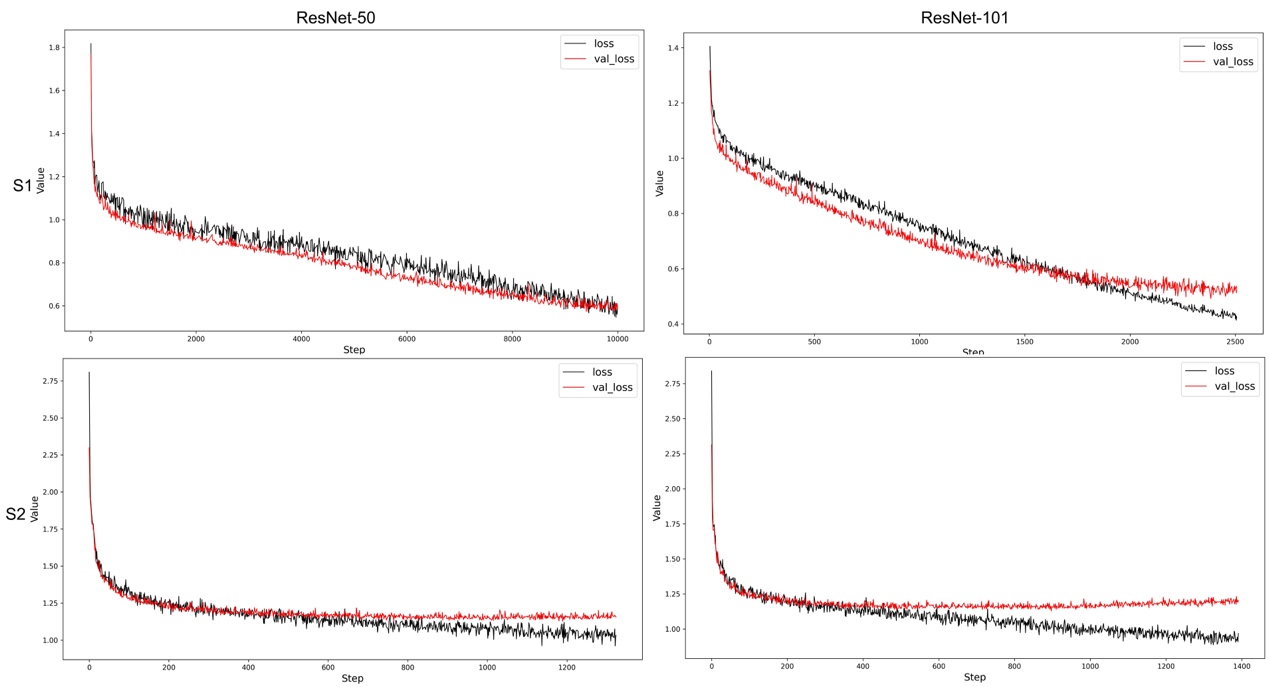


Figure S1 Details of the training of the Mask R-CNN network.

Table S1 Comparison of mA（IOU=50）for Mask R-CNN of different backbone, YOLOACT++ and SOLOv2 models.

| Study sites | | Mask R-CNN(ResNet-101) | Mask R-CNN(VGG) | Mask R-CNN(Inception) | YOLOACT++(ResNet-101) | | SOLOV2(ResNet-101) |
| --- | --- | --- | --- | --- | --- | --- | --- |
| S1 | 98.32±1.06 | | 76.69±0.89 | 77.95±1.24 | 75.82±0.83 | 81.23±1.57 | |
| S2 | 85.81±2.26 | | 74.11±1.34 | 71.96±1.32 | 66.46±0.62 | 76.39±1.13 | |
